# Performance Analysis of ToA-Based Positioning Algorithms for Static and Dynamic Targets with Low Ranging Measurements

**DOI:** 10.3390/s17081915

**Published:** 2017-08-19

**Authors:** André G. Ferreira, Duarte Fernandes, André P. Catarino, João L. Monteiro

**Affiliations:** 1 Algoritmi Center, University of Minho, 4800-058 Guimarães, Portugal; id4542@alunos.uminho.pt (D.F.); joao.monteiro@dei.uminho.pt (J.L.M.); 2 Center of Textile Science and Technology, University of Minho, 4800-058 Guimarães, Portugal; whiteman@det.uminho.pt

**Keywords:** algorithms comparison, emergency responders, indoor positioning system (IPS), NLOS identification and mitigation, positioning algorithms, unstructured environments, UWB

## Abstract

Indoor Positioning Systems (IPSs) for emergency responders is a challenging field attracting researchers worldwide. When compared with traditional indoor positioning solutions, the IPSs for emergency responders stand out as they have to operate in harsh and unstructured environments. From the various technologies available for the localization process, ultra-wide band (UWB) is a promising technology for such systems due to its robust signaling in harsh environments, through-wall propagation and high-resolution ranging. However, during emergency responders’ missions, the availability of UWB signals is generally low (the nodes have to be deployed as the emergency responders enter a building) and can be affected by the non-line-of-sight (NLOS) conditions. In this paper, the performance of four typical distance-based positioning algorithms (Analytical, Least Squares, Taylor Series, and Extended Kalman Filter methods) with only three ranging measurements is assessed based on a COTS UWB transceiver. These algorithms are compared based on accuracy, precision and root mean square error (RMSE). The algorithms were evaluated under two environments with different propagation conditions (an atrium and a lab), for static and mobile devices, and under the human body’s influence. A NLOS identification and error mitigation algorithm was also used to improve the ranging measurements. The results show that the Extended Kalman Filter outperforms the other algorithms in almost every scenario, but it is affected by the low measurement rate of the UWB system.

## 1. Introduction

During last decade, great progress has been made on the development of Indoor Positioning Systems (IPSs). Both academia and industry are targeting high-accurate IPSs for a variety of applications and scenarios. It is commonly accepted in both communities that the positioning of emergency responders during their missions is one of the most challenging scenarios for an IPS [1,2,3,4,5,6,7,8]. The missions’ scenarios are unstructured, cover wide operative areas and the surrounding environment is harsh and highly dynamic. All these application specific constraints make the use of preinstalled infrastructure, maps and localization method that require an offline or calibration phase (e.g., fingerprinting) unfeasible [2,5,7,8].

Currently, based on the technological principle, the IPSs for emergency responders can be classified as radio signal-based, IMU-based and hybrid systems [8]. Radio signal-based IPSs can be designed based on Wi-Fi [5,9,10,11], ultra-wide band (UWB) [12,13], ZigBee [14,15], Bluetooth [9,16] and RFID [9,16]. These can be used alone or combined to improve the IPS accuracy due to the different granularities that each technology provides. The main advantages of these systems are: the capabilities of radios waves to travel through obstacles, the system performance is not affected by the user’s motion (e.g., walking, running, and crawling) and can be improved by deploying more nodes on the scenario, and the positioning infrastructure can be reused for communication [8]. Whereas, their disadvantages are as follows: requires at least three different ranging measurements to compute the user’s position, the interference with the emergency responders’ operations (they have to deploy nodes as they enter a building), the performance degradation due to the radio propagation phenomena (e.g., non-line-of-sight NLOS conditions, high temperatures, thick smoke and humidity), and the risk of some anchor nodes being destroyed by the fire or falling debris [8].

IMU-based IPSs are another area that has attracted many researchers to address the problem of localization for emergency responders [4,7,17,18,19,20,21,22,23,24]. These systems are based on inertial and motion sensors (e.g., 3D accelerometer, 3D gyroscope, 3D magnetometer, and barometer) that compose an inertial measurement unit (IMU). These IMUs can be mounted on the head [17,20], chest [4], foot [19,21], dual foot [23,24] and different body segments [7,18,22]. The advantages of such IPSs are: zero radiation signature, low-cost, they do not require additional infrastructure, they provide continuous positioning, and are capable of operating in all indoor environments [8]. The drawbacks of these systems are that the error grows quickly as the time interval without position correction increases, the performance of the IPS is affected by the type of the movement, and they do not have a communication infrastructure to send the computed position to the incident commander [8].

Finally, the hybrid systems combine both technologies (radio and IMU) to overcome the limitations when each technology is used alone. A data fusion algorithm is used to merge the positioning data obtained from both subsystems. Relate Trails [25], PPL [26], Virtual Lifeline [27], GLANSER [28], WASP [29], and the work of Simon et al. [30] are examples of hybrid systems for emergency responders. The advantages of these systems are: improved accuracy, continuous position estimation, bidirectional communication, adaptability to all environments, and immunity to the user’s motion [8]. However, these IPSs also have some drawbacks, namely: increased complexity and development time due to the data fusion algorithms, higher cost and energy consumption, and a radiation signature [8].

In this paper, the UWB-based IPS of the PROTACTICAL Personal Protective Equipment (PPE) is presented and discussed. The PROTACTICAL PPE is a project financed by the Portuguese QREN program (I&IDT-Project in Co-promotion No. 23267), which aims to improve the emergency responder’s performance, resilience, and safety. Besides monitoring the position of the emergency responders, the PROTACTICAL PPE also provides thermal isolation, monitoring of physiological and environmental parameters, and real-time communication between the emergency responder and the incident commander. When compared with other localization technologies, the UWB is capable of providing robust signaling, through-wall propagation and provides a large bandwidth that allows high-resolution ranging even in harsh environments, which makes it an attractive solution for emergency responders’ IPSs [7,31,32,33,34]. Typically, the UWB transceivers rely on the Time-of-Flight (ToF) technique for the ranging estimation. However, in indoor environments, these estimates are likely to be affected by NLOS propagation, which leads to positive biases in distance estimation [35,36]. Due to the unstructured nature of the environments where the missions of emergency responders take place, it is very difficult to predict which obstruction caused the NLOS propagation. The only assumption that can be made is that the human body can lead to it, since the UWB transceiver is mounted on the user [31,32,33,37].

Emergency responder’s missions represent a challenging indoor positioning application that imposes strict requirements on the design of the IPS. Therefore, the goal of this paper is to evaluate and compare different positioning algorithms and select the one that best suits in such scenario. So, based on the IPS requirements defined in [8], the performance assessment of the algorithms is conceived as follows:

- High Performance with Low Ranging Measurements—unlike Wireless Sensor Networks (WSNs) applications, where tens of ranging measurements can be available [38,39,40,41], during emergency responders’ missions the availability of radio signals is generally low. This happens due to the following reasons: no reliable infrastructure exists in a building capable of computing the emergency responders’ position, the deployment cannot interfere with the emergency responder’s activities, the low penetration capability of UWB signals in indoor environments (up to 40 m in NLOS scenarios), and the risk of some anchor nodes being destroyed by the fire or falling debris. So, the performance (accuracy, precision and root mean square error (RMSE)) of the positioning algorithms is assessed with only three ranging measurements. This is the minimum number of measurements required to compute the user’s position;
- Information Accessibility—the position update rate of the UWB-based IPS is between 1 and 2 Hz, which clearly comply with the requirements (<40 s) [8,11]. Due to the nature of the UWB technology, the information security is also guaranteed;
- Immunity to Environment-Related Perturbations—the performance of the system must be independent of the scenario, so the positioning algorithms are tested under different scenarios (atrium and lab), propagation conditions (LOS and NLOS due to a human body) and movement (static and dynamic). In other words, we want to evaluate which positioning algorithm presents higher immunity to noisy measurements and, therefore, is more likely to provide a robust position estimation under the different propagation conditions of indoor environments. The performance of the localization algorithms is also compared after running a NLOS identification and error mitigation algorithm developed for the NLOS caused by the human body. This method proved to be an effective way to improve the performance of all algorithm under NLOS condition.

Although this work is conducted within the scope of an IPS for emergency responders, the work here performed and its conclusions are transversal to other indoor positioning applications. The remainder of the paper is organized as follows: Section 2 presents the materials and methods used to perform and validate this research. In this section, the UWB transceivers used to acquire the ranging measurements, the NLOS identification and error mitigation algorithm for UWB transceivers mounted on the human body, the localization algorithms, the performance metrics, and the experimental setup are described. The results and the discussion of the experiments are presented in Section 3 and Section 4, respectively. Finally, Section 5 presents the conclusions of the work performed.

## 2. Materials and Methods

### 2.1. DW1000 UWB Transceiver

The DW1000 chip is a UWB transceiver compliant with the IEEE802.15.4-2011 standard developed by DecaWave (Dublin, Ireland) that allows very accurate ranging measurements [42]. The main advantage of this UWB transceiver when compared with its competitors (e.g., Ubisense and Time Domain products) is the low cost of each transceiver (approx. €19 per unit). These transceivers can transmit pulses that are few nanoseconds long with a bandwidth of 500 or 900 MHz and a frequency center that spans from 3.5 to 6.5 GHz. The high temporal resolution required to perform UWB communication allows an accuracy of the ranging measurements down to a few centimeters in line-of-sight (LOS) conditions [42]. Due to its high bandwidth and spectrum usage, the transmit power density of the UWB transceivers is limited to −41.3 dBm/MHz to avoid inter-system interference. This restriction limits the operational range of the UWB transceivers, up to 300 m in LOS and 40 m in NLOS [42].

The ranging measurements in these transceivers is performed based on the two way ranging (TWR) that relies on ToF technique. The tag node is responsible for starting the ranging procedure and the anchor for computing the respective distance. In this work, the DW1000 UWB transceivers are configured to operate on channel 4 (500-MHz bandwidth with a center frequency of 3993.6 MHz), preamble length of 1024 and a data rate of 110 kb/s.

### 2.2. NLOS Identification and Error Mitigation

This section is a summary of work conducted by the authors that is to be published separately and is currently under review. Full details of the measurement campaign and the NLOS identification and error mitigation algorithm are detailed in [43].

A key feature of the DW1000 UWB transceivers is their built-in diagnostic capability. Through the processing of the CIR data obtained from the received waveforms, it is possible to infer the propagation condition (LOS or NLOS). Common metrics to assess the channel condition are: amplitude (e.g., RSS, maximum amplitude of the received signal, power difference and power ratio), temporal (e.g., ToA, RMS delay-spread, peak-to-lead delay, rise time, mean excess delay and maximum excess delay), and CIR data distribution (kurtosis and skewness) [35,44,45]. The amplitude-based statistics are immediately, or with little processing, available from the DW1000 UWB transceivers, whereas the temporal and CIR data distribution statistics require an additional processing that can add a delay of 4–5 s [44]. This additional delay can compromise the real-time requirements of IPS for emergency responders.

A measurement campaign was conducted in a corridor to evaluate the impact that different propagation conditions have on the amplitude-based statistics and to determine which is better for NLOS identification. During this measurement campaign four different propagation conditions were assessed, one LOS and three NLOS (caused by a fire door, a wall, and the human body). For each test, the distance between the two transceivers varies from 1 up to 44 m. Based on the results obtained, it was verified that highest ranging error is obtained when the UWB transceiver is mounted on the human body and the best metric for NLOS identification is the power difference ($P_{D}$). $P_{D}$ is defined as follows:

(1) $$P_{D}=\;P_{T}-P_{FP}$$

where $P_{T}$ and $P_{FP}$ are, respectively, the estimated received power and the RSS in the first path, which are defined as follows [46]:

(2) $$P_{T}=10\times \log_{10}\left(\frac{C\times 2^{17}}{N^{2}}\right)-A$$

(3) $$P_{FP}=10\times \log_{10}\left(\frac{F_{1}{}^{2}+\;F_{2}{}^{2}\;+\;F_{3}{}^{2}}{N^{2}}\right)-A$$

where C is the channel impulse response power, N is the preamble accumulation count, A is a system predefined constant (121.74 dBm for a pulse repetition frequency of 64 MHz), and $F_{1}$, $F_{2}$, and $F_{3}$ are the first path amplitude points. All these parameters are acquired from the registers of the DW1000 UWB transceiver after the reception of a ranging message.

According to the results of the measurement campaign under the human body influence, the $P_{D}$ metric is uncorrelated with the true distance and has a low overlap between the LOS and NLOS condition. So, a simple threshold based algorithm was implemented for NLOS identification:

(4) $$\{\begin{array}{c} P_{D}>th_{P_{D}},\;NLOS\;condition\\ P_{D}<th_{P_{D}},\;LOS\;condition \end{array}$$

where is the static threshold that minimizes the misclassification rate. An accuracy of 93% was obtained in the identification of the NLOS condition caused by the human body.

For the NLOS error mitigation, since it varies with the distance, a second-order polynomial model was proposed:

(5) $$f\left(\hat{d}\right)=\;p_{1}\times {\hat{d}}^{2}+p_{2}\times \hat{d}+p_{3}$$

where $f\left(\hat{d}\right)$ is the estimated ranging error, $\hat{d}$ is the estimated distance, and $p_{1}$, $p_{2}$, and $p_{3}$ are the curve specific parameters. This model was obtained by calculating the median of the estimated distances—for each evaluated distance—and the NLOS error—measured in the NLOS dataset with the human body influence. With the proposed NLOS error mitigation the standard deviation of the ranging measurements was reduced by 60% (from 3.72 to 1.47 m), and the ranging error was successfully approximated to a white Gaussian distribution. Table 1 shows the parameters values for the proposed NLOS identification and error mitigation algorithm. These values were obtained from the curve fitting toolbox of Matlab and are used during the experimental evaluation.

### 2.3. ToA-Based Localization Algorithms

In this subsection, four typical ToA-based localization algorithms are introduced. All the analyzed algorithms rely on ranging measurements, provided by the DW1000 UWB transceiver, to compute the 2D localization of the tag node. To keep the complexity of the localization algorithm low, the tag’s height is not computed by the localization algorithm. As an alternative, it can be obtained from additional sensors like barometers or pressure sensors. However, the extension to 3D is straightforward for all proposed algorithms. For simplicity, the position of the anchor nodes is known and does not change during experiments.

The four ToA-based localization algorithms studied are: the analytical method, the least-squares method, the nonlinear least-squares method based on a first-order Taylor expansion (Taylor series), and the EKF. Each algorithm has different complexity and is designed to address different issues on localization. The first two algorithms are the simplest to implement and their differences lie in scalability and flexibility. For the analytical method, the number of possible ranging measurement combinations has to be known beforehand since one equation has to be defined for each tag-anchor pair. On the other hand, the least-squares method allows adding more tag-anchor pairs without having to rewrite the algorithm. Both algorithms do not handle with the covariance of the ranging measurements error. To deal with the nonlinearity issue aroused by the localization problem and the covariance of the ranging measurements, both the nonlinear least-squares method based on a first-order Taylor expansion and the EKF are proposed. Although the complexity of these algorithms is higher, it is expected to observe an improvement in performance when compared with the first two algorithms. While the Taylor series is an extension to the trilateration-based localization algorithms, the EKF is a predictive algorithm that aims to predict the next state based on a system model and the ranging measurements.

For the trilateration-based localization algorithms, the position of the tag is determined as the intersection of all circles. The center and radius of each imaginary circle are given by the coordinates of the corresponding anchor node and the ranging measurement between that anchor and the tag, respectively. Therefore, the circles can be described as:

(6) $${\left(x-x_{i}\right)}^{2}+{\left(y-y_{i}\right)}^{2}=d_{i}^{2},\;\left(i=1,2,\ldots ,n\right)$$

where (x,y) is the position of the tag, $\left(x_{i},y_{i}\right)$ is the known position of anchor i, n is the number of anchor nodes, and $d_{i}$ is the true distance between anchor i and the tag. The value of $d_{i}$ is obtained by applying the following equation:

(7) $$\hat{d}=c\times ToA=\{\begin{array}{c} d+e_{LOS},\;\mathrm{if}\;\mathrm{LOS}\;\\ d+\;e_{NLOS},\;\mathrm{if}\;\mathrm{NLOS} \end{array}=\{\begin{array}{c} d+\;e_{LOS},\;\mathrm{if}\;\mathrm{LOS}\;\\ d+\;e_{LOS}+b,\;\mathrm{if}\;\mathrm{NLOS} \end{array}$$

where $c=3\times 10^{8}$ is the speed of light, ToA is the reported Time of Arrival, d is the true distance between the transmitter and the receiver, $e_{LOS}$ is the ranging error in the LOS scenario, which includes all typical sources of error of a UWB ranging system (i.e., finite bandwidth, clock drift, PCB losses, thermal noise, etc.), and $e_{NLOS}$ is the result of $e_{LOS}$ ranging error with the positive and random bias b caused by multipath propagation in the NLOS scenario.

As demonstrated in the previous subsection, the accuracy of range estimation $d_{i}$ is affected by several phenomena (e.g., noise, multipath, fading to ground-bounce, and NLOS). If the ranging error is additive, this results that the circles will not intersect at one single point. On the other hand, if the ranging error is subtractive, the circles may not intersect. So, the goal of the localization algorithm is to estimate the tag position as close as the true tag position, even in the presence of noisy measurements.

#### 2.3.1. Analytical Method

The analytical method is the simplest localization algorithm. This method determines the tag position by solving the nonlinear equations directly [39,47,48,49].

So, for the scenario when only three anchor nodes are available, which is the minimum number of different ranging measurements required, the localization problem is a set of three equations with two unknowns:

(8) $$\{\begin{array}{c} {\left(x-x_{1}\right)}^{2}+{\left(y-y_{1}\right)}^{2}=d_{1}^{2}\\ {\left(x-x_{2}\right)}^{2}+{\left(y-y_{2}\right)}^{2}=d_{2}^{2}\\ {\left(x-x_{3}\right)}^{2}+{\left(y-y_{3}\right)}^{2}=d_{3}^{2} \end{array}$$

Different techniques were proposed to solve the nonlinear equations above. In this work, the linear algorithm proposed in [49] is used as the analytical method. This method computes the position of the tag by the intersection of two virtual lines created from the two points where the imaginary circles intersect. So, for an IPS with only three anchor nodes, the line that passes through the intersection of the two circles (e.g., the circles centered at anchors 1 and 2) can be found by differencing the corresponding ranges in (8). The resulting equation is:

(9) $$\left(x_{2}-x_{1}\right)x+\left(y_{2}-y_{1}\right)y=\frac{1}{2}\left(\|A_{2}{\|}^{2}-\|A_{1}{\|}^{2}+d_{1}^{2}-d_{2}^{2}\right)$$

where $\left\|A_{i}\right\|$ is the norm of the position of anchor i.

If the same procedure is repeated for anchors 2 and 3, the following line equation is obtained:

(10) $$\left(x_{3}-x_{2}\right)x+\left(y_{3}-y_{2}\right)y=\frac{1}{2}\left(\|A_{3}{\|}^{2}-\|A_{2}{\|}^{2}+d_{2}^{2}-d_{3}^{2}\right)$$

A new line can be created for anchors 1 and 3. However, this line is not independent of the above lines, i.e., does not add useful information about the tag position since the three lines will always intersect in the same point [49]. So, for 2D localization only two lines are needed.

The position of the tag is obtained by solving (9) and (10) in terms of y, equating the obtained results, and solving in terms of x. The resulting equation is:

(11) $$x=\frac{\left(y_{2}-y_{1}\right)C_{3}-\left(y_{3}-y_{2}\right)C_{1}}{\left[\left(x_{3}-x_{2}\right)\left(y_{2}-y_{1}\right)-\left(x_{2}-x_{1}\right)\left(y_{3}-y_{2}\right)\right]}$$

where:

(12) $$C_{1}=\frac{1}{2}\left(\|A_{2}{\|}^{2}-\|A_{1}{\|}^{2}+d_{1}^{2}-d_{2}^{2}\right)$$

(13) $$C_{3}=\frac{1}{2}\left(\|A_{3}{\|}^{2}-\|A_{2}{\|}^{2}+d_{2}^{2}-d_{3}^{2}\right)$$

By substituting (11) into either (9) or (10) and solving in terms of y, gives:

(14) $$y=\frac{\left(x_{2}-x_{1}\right)C_{3}-\left(x_{3}-x_{2}\right)C_{1}}{\left[\left(y_{3}-y_{2}\right)\left(x_{2}-x_{1}\right)-\left(y_{2}-y_{1}\right)\left(x_{3}-x_{2}\right)\right]}$$

An important consideration about this method is that the two lines may not intersect due to ranging errors or due to the geometric distribution of the anchors. In such scenario, the position of the tag cannot be computed.

#### 2.3.2. Least-Squares Method

The nonlinear equations in (8) can be expressed in a matrix form after some mathematical manipulation. So, they can be written as [47]:

(15) $$At=\frac{1}{2}b$$

where:

(16) $$A=\left[\begin{array}{c} \begin{array}{ccc} x_{1} & y_{1} & -0.5 \end{array}\\ \begin{array}{ccc} x_{2} & y_{2} & -0.5 \end{array}\\ \ldots \\ \begin{array}{ccc} x_{n} & y_{n} & -0.5 \end{array} \end{array}\right]$$

(17) $$t={\left[x\;y\;s\right]}^{T}$$

(18) $$b=\left[\begin{array}{c} k_{1}-d_{1}^{2}\\ k_{2}-d_{2}^{2}\\ \begin{array}{c} \ldots \\ k_{n}-d_{n}^{2} \end{array} \end{array}\right]$$

and:

(19) $$k_{i}=x_{i}^{2}+y_{i}^{2},\;\left(i=1,2,\ldots ,n\right)$$

(20) $$s=x^{2}+y^{2}$$

To avoid the quadratic parameter s, Caffery proposed an alternative method for cancelling out the nonlinear terms and producing a linear model [49]. This method works by selecting on an equation in (8) (e.g., i=1) and subtracting it from the other equations. However, the accuracy of this method is highly dependent on the distance from the selected anchor node and the tag, deteriorating as the distance between these nodes increase [47]. So, to keep the complexity of the localization algorithm low, it was selected the traditional least-squares method.

#### 2.3.3. Nonlinear Least-Squares Method based on a First-Order Taylor Expansion (Taylor Series)

A common strategy to linearize the nonlinear function $d_{i}\left(X\right)$ in (8) around a reference point $X_{0}$ is to use the Taylor series expansion. If an initial estimation of the position is available $\left(x_{0},y_{0}\right)$ and the higher terms are neglected, the function $d_{i}\left(X\right)$ can be expressed as [39,47,50]:

(21) $$d\left(X\right)\approx d\left(X_{0}\right)+H_{0}\left(X-X_{0}\right)$$

where $X_{0}$ is the vector of the initial estimation, X is the vector of the anchor nodes’ coordinates, and $H_{0}$ represents the Jacobian matrix of d(X) around $X_{0}$, which can be represented as:

(22) $$H_{0}={\left[\begin{array}{c} \begin{array}{cc} \frac{\partial d_{1}}{\partial x} & \frac{\partial d_{1}}{\partial y} \end{array}\\ \begin{array}{cc} \frac{\partial d_{2}}{\partial x} & \frac{\partial d_{2}}{\partial y} \end{array}\\ \ldots \\ \begin{array}{cc} \frac{\partial d_{n}}{\partial x} & \frac{\partial d_{n}}{\partial y} \end{array} \end{array}\right]}_{X=X_{0}}=\left[\begin{array}{c} \begin{array}{cc} \frac{x_{0}-x_{1}}{r_{1}} & \frac{y_{0}-y_{1}}{r_{1}} \end{array}\\ \begin{array}{cc} \frac{x_{0}-x_{2}}{r_{2}} & \frac{y_{0}-y_{2}}{r_{2}} \end{array}\\ \ldots \\ \begin{array}{cc} \frac{x_{0}-x_{n}}{r_{n}} & \frac{y_{0}-y_{n}}{r_{n}} \end{array} \end{array}\right]$$

This assumption is only valid if the initial estimation is sufficiently close to the true location of the tag node. The initial position estimation is computed based on the analytical method proposed above. The value of $r_{i}$ is obtained as following:

(23) $$r_{i}=\sqrt{{\left(x_{0}-x_{i}\right)}^{2}+{\left(y_{0}-y_{i}\right)}^{2}}$$

Equation (21) can be written in matrix form as:

(24) $$H_{0}\delta =M-e$$

where:

(25) $$M=\left[\begin{array}{c} {\hat{d}}_{1}-d_{1}\\ {\hat{d}}_{2}-d_{2}\\ \ldots \\ {\hat{d}}_{n}-d_{n} \end{array}\right]$$

(26) $$\delta =\left[\begin{array}{c} \delta_{x}\\ \delta_{y} \end{array}\right]$$

(27) $$e=\left[\begin{array}{c} \varepsilon_{1}\\ \varepsilon_{2}\\ \ldots \\ \varepsilon_{n} \end{array}\right]$$

and $\varepsilon_{i}$ represents the range estimation error. The mean and variance of range estimation error are defined according the channel state (LOS or NLOS).

Since the ranging measurements are independent and its error follow a Gaussian distribution, the weighted least squares solution of (24), with respect to δ, can be determined based on the maximum likelihood (ML) estimation and is given as [51,52]:

(28) $$\delta ={\left(H_{0}{}^{T}R^{-1}H_{0}\right)}^{-1}H_{0}{}^{T}R^{-1}M$$

where R is the covariance matrix of the estimation error e, whose terms are independent and zero-mean Gaussian random variables, and can be represented as:

(29) $$R=E\left\{\varepsilon \varepsilon^{T}\right\}=diag\left[\begin{array}{ccc} \sigma^{2} & \ldots & \sigma^{2} \end{array}\right]$$

The value of $\sigma^{2}$ was experimentally determined during the measurement campaign described in Section 2.2. This value has been calculated based on the mean of the variances calculated for each measurement point. A $\sigma^{2}=0.02^{2}$ is used for the experimental evaluation.

Based on the initial position estimation $\left(x_{0},y_{0}\right)$ and the computed δ, the position estimation can be updated as follows:

(30) $$\{\begin{array}{c} x=x_{0}+\delta_{x}\\ y=y_{0}+\delta_{y} \end{array}$$

By iterating the above process, the position estimation can be repeatedly refined. The process is repeated until the convergence is achieved, i.e., $\delta_{x}$ and $\delta_{y}$ turn out to be satisfactorily small according to some criterion, or the maximum iterations are achieved [39,47,50]. The final position estimation is defined based on the position whose convergence criterion was minimum. In this paper the convergence criterion is:

(31) $$\Phi =M^{T}R^{-1}M$$

This convergence criterion represents the sum of the square error between the Euclidean distance estimates of the previous and current position. Each distance is weighted according to the covariance matrix R. So, if a measurement is taken in NLOS, the uncertainty (covariance) of that measurement is higher and, therefore, it will have a higher impact on the value of Φ.

#### 2.3.4. Extended Kalman Filter Method

Unlike the previous localization algorithms, the EKF is tailored for tracking mobile nodes. Its main advantage is that the EKF can process single measurements at a time and provide position estimates in real-time. The EKF performance highly depends on the correct definition of the system dynamics [41]. Based on the information acquired from the motion (e.g., velocity, acceleration, angular velocity), different EKF formalizations can be made to model the movement of a person. A commonly used model for pedestrians is to model the movement of the mobile device as random. This simple model has proven to be more robust than other complex models because of the fact that human’s movement is unpredictable and, therefore, better modeled as Gaussian noise. Other alternative are models that include the velocity, velocity and acceleration, and orientation.

In this work, the random model was selected to describe the pedestrian movement. With this model, the changes in position are given by Gaussian noise. Therefore, the state transition model of the system can be defined as:

(32) $$X_{k+1}=AX_{k}+w_{k}$$

where $X_{k+1}$ and $X_{k}={\left[\begin{array}{cc} x_{tag} & y_{tag} \end{array}\right]}^{T}$ represent, respectively, the current and the previous position state vectors. $w_{k}$ is the process noise that allows changes in position and orientation with covariance matrix $Q_{k}={\left[\begin{array}{cc} \epsilon_{x} & \epsilon_{y} \end{array}\right]}^{T}$. The values of the covariance matrix $Q_{k}$ were determined empirically. The matrix A represents the state transition matrix and is modeled as an identity matrix:

(33) $$A=I_{2}=\left[\begin{array}{c} \begin{array}{cc} 1 & 0 \end{array}\\ \begin{array}{cc} 0 & 1 \end{array} \end{array}\right]$$

The measurement model can be represented by:

(34) $$Z_{k}=h\left(X_{k}\right)+v_{x}$$

where $Z_{k}$ is the current ranging measurements vector, $h\left(X_{k}\right)$ is the observation matrix, and $v_{x}$ is the measurement noise whose covariance is $R_{k}$. The index k indicates that the parameters can change over time. The observation matrix $h\left(X_{k}\right)$ and the corresponding Jacobian $H_{k}$ are derived from (8) and are given as follows:

(35) $$h\left(X_{k}\right)=\sqrt{{\left(x-x_{k}\right)}^{2}+{\left(y-y_{k}\right)}^{2}}$$

(36) $$H_{k}=\left[\begin{array}{cc} \frac{x-x_{k}}{\sqrt{{\left(x-x_{k}\right)}^{2}+{\left(y-y_{k}\right)}^{2}}} & \frac{y-y_{k}}{\sqrt{{\left(x-x_{k}\right)}^{2}+{\left(y-y_{k}\right)}^{2}}} \end{array}\right]$$

Based on the models described above, the EKF estimates the tag position based on two different stages: prediction and update:

Prediction Stage:

During the prediction stage, the EKF predicts the state vector ($X_{k}^{-}$) and error covariance matrix ($P_{k}^{-}$), which are given as follows:

(37) $$X_{k}^{-}=AX_{k-1}=X_{k-1}$$

(38) $$P_{k}^{-}=AP_{k-1}A^{T}+Q_{k}$$

Update Stage:

As soon as new ranging measurements are available ($Z_{k}$), the update stage can be applied. This stage aims to refine the state vector ($X_{k}^{-}$) and the error covariance $(P_{k}^{-}$) estimates. The first step of the update stage is computing the Kalman gain. The Kalman gain is the ratio between the uncertainty of the prediction and the uncertainty of the measurements, and is computed as follows:

(39) $$K_{k}=P_{k}^{-}H_{k}^{T}S_{k}^{-1}$$

where $H_{k}^{T}$ is the transpose matrix of the observation matrix $H_{k}$ and $S_{k}^{-1}$ is the inverse of the residual covariance matrix $S_{k}$ that is computed as follows:

(40) $$S_{k}=H_{k}P_{k}^{-}H_{k}^{T}+R_{k}$$

Then, the Kalman gain is used to combine the received ranging measurement information with the information from the prediction stage in order to compute the update state as follows:

(41) $$X_{k}=X_{k}^{-}+R_{k}{\tilde{y}}_{k}$$

where:

(42) $${\tilde{y}}_{k}=Z_{k}-h\left(X_{k}^{-}\right)$$

is known as innovation or measurement residual, and $h\left(X_{k}^{-}\right)$ represents the predicted measurements. In terms of EKF performance, lower values of innovation or Kalman gain are desirable, i.e., small values of innovation or Kalman gain imply small corrections in the predicted state and, therefore, a smoother tracking system. The last step of the EKF is the update of the error covariance as follows:

(43) $$P_{k}=\left(I-K_{k}H_{k}\right)P_{k}^{-}$$

where I in an identity matrix with appropriate dimensions.

For static devices, however, the predicted state vector ($X_{k}^{-}$) and the predicted covariance matrix ($P_{k}^{-}$) are expected to remain unchanged between measurements. So, for static devices, the covariance matrix $Q_{k}$ is removed from Equation (38). The update stage is the same as for mobile devices.

### 2.4. Performance Evaluation Metrics

Performance metrics provide the basis for comparing localization algorithms [53]. So, the performance of the above positioning algorithms under the different scenarios is compared based on the following metrics: accuracy, precision, and Root Mean Square Error (RMSE).

Traditionally, the accuracy is represented by the mean distance error and the precision is defined as the success probability of position estimates with respect to the accuracy [53,54]. However, this strategy lacks to provide useful information about an IPS’s precision, since the precision is always associated with the accuracy and these metrics are independent. So, both accuracy and precision are presented as a cumulative distribution function (CDF) and they are expressed as a value for a specific percentage (e.g., an accuracy of 1.6m with 95% probability).

#### 2.4.1. Accuracy

The accuracy is the most used metric to evaluate the performance of a positioning algorithm or IPS. It represents the difference between the true position and the estimated position. This metric is generally measured as the Euclidean distance between the estimated position and the true position, as defined by the following equation:

(44) $$D_{Accuracy}=\sqrt{{\left(x_{Est}-x_{Actual}\right)}^{2}+{\left(y_{Est}-y_{Actual}\right)}^{2}}$$

where $\left(x_{Est},y_{Est}\right)$ are the Cartesian coordinates estimated by the localization algorithm, and $\left(x_{Actual},y_{Actual}\right)$ are the true Cartesian coordinates.

#### 2.4.2. Precision

The precision measures the reproducibility of successive position estimates. This metric can be used to assess the robustness of the positioning algorithm as it reveals the variation of position estimates over several trials [53]. To compute the precision, we first compute the median position of the 200 position estimations for a single test run. Then, the Euclidean distance to each estimated position is computed based on the median position. The precision is computed based on the following equation:

(45) $$D_{Precision}=\sqrt{{\left(x_{Est}-x_{median}\right)}^{2}+{\left(y_{Est}-y_{median}\right)}^{2}}$$

#### 2.4.3. Root Mean Square Error (RMSE)

Unlike the accuracy metric, the RMSE metric allows computing the localization error for both X and Y coordinates. The RMSE value per each coordinate can be computed by:

(46) $$RMSE_{i}=\sqrt{\frac{\sum^{\text{​}}{\left(Est_{i}-Actual_{i}\right)}^{2}}{Number\;of\;Estimates}}$$

where i represents the coordinate axis.

The $RMSE_{X}$ and $RMSE_{Y}$ values can be combined to compute the Net RMSE, which is the net error of the localization algorithm. The RMSE values are biased towards large errors, i.e., a large error makes a larger contribution in RMSE than in a simple average. The Net RMSE can be computed as following:

(47) $$Net\;RMSE=\sqrt{RMSE_{X}{}^{2}+RMSE_{Y}{}^{2}}$$

### 2.5. Experimental Setup

In this section, we describe the deployment scenarios used to evaluate the performance of the algorithms described above. In these experiments, the DW1000 UWB transceivers, already described in Section 2.1, are used to collect the ranging measurements needed to run the localization algorithms. Two types of nodes are considered, anchor and tag nodes. Both nodes are identical in terms of hardware. The anchor nodes are placed on a tripod at an antenna height of 1.33 m and their position is known. The tag is responsible for starting the ranging message with an anchor node, computing the corresponding distance between the nodes, acquiring the channel propagation parameters necessary for NLOS identification and mitigation, and logging this data to a computer through a USB connection. The tag node repeats this process continuously for all anchor nodes available, starting from anchor 1 to anchor n. Where n is the number of anchor nodes available. This cycle is repeated until all the samples per point are collected, or the user completes the predefined path. All the localization algorithms are implemented in MATLAB, run offline, and use the same data set. In this way, we guarantee that all the algorithms are evaluated under the same conditions and, therefore, a fair comparison can be performed.

**Figure 1:**
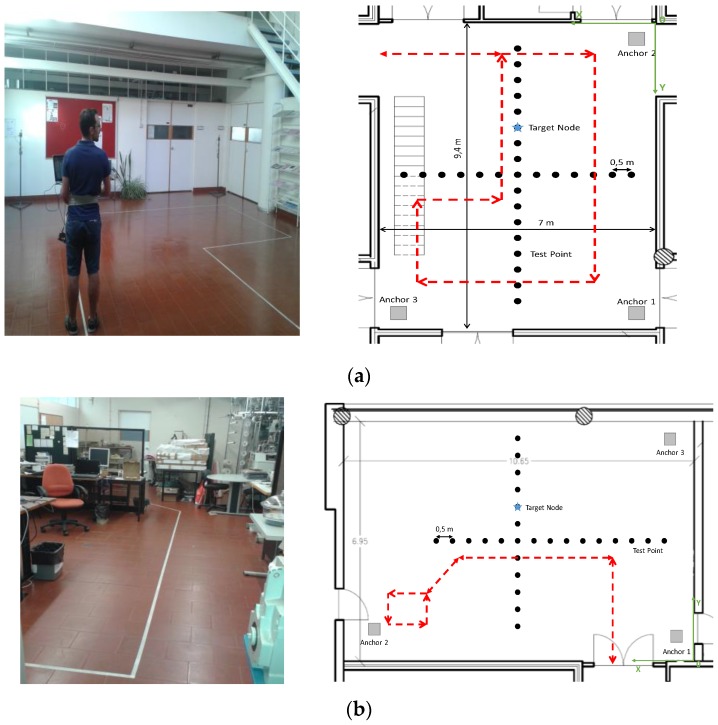
Illustration of the experimental setup in: (**a**) atrium; (**b**) lab.

Figure 1 illustrates the two test beds considered to evaluate the localization algorithms. The two scenarios are, respectively, an atrium with 9.4 m× 7 m free space area (Figure 1a), and a lab with an area of 10.7 m × 7 m, desks, metallic cabinets, and textile machines (Figure 1b).

The gray squares represent the location of anchor nodes, the blue star represents an example of the tag location, and the black dots are the calibration points evaluated for the static scenarios. The position of the calibration points was acquired based on a digital laser rangefinder. The red line represents the path performed during the dynamic test. The main goal of considering these two environments is to assess how different propagation characteristics affect the performance of each algorithm. In other words, we want to verify which algorithm has higher immunity to environment-related perturbations. For each scenario described above, three sets of measurements were conducted: static without body interference (Case 1), where the tag is placed on the top of a tripod at an antenna height of 1.33 m, static under body influence (Case 2), where the tag was mounted next the right side of the waist of the human body at an antenna height of 1.08 m; and dynamic (Case 3), where the tag was mounted next the right side of the waist of the human body at an antenna height of 1.08 m and the user walks through a predefined path. For Cases 2 and 3 an additional distance correction is performed before run the NLOS identification and mitigation algorithm. This distance correction aims to correct the distance error due to the difference in heights between anchors and tag when the tag is mounted on the human body. The calibration points of the both scenarios were taken in a cross form, centered at the middle of the scenario, and with a spacing between points of 0.50 m. We choose this configuration because of machines and desks in the lab scenario, which does not allow us to collect a grid of points evenly distributed. Nevertheless, with this approach the performance of each algorithm in both x and y directions can be assessed, both scenarios can be easily compared, and the measurement campaign took less time. For each evaluated point in the static measurements, 200 samples were collected per anchor node. Whereas, in the dynamic case the experiment was run five times. The goal of this experiments is to distinguish between device-related effects (e.g., clock drift, antenna placement, and radiation pattern) and body effects, as well as, between static and dynamic situations. During the experiments no other people were allowed to stay or walk through the scenarios.

## 3. Results

In this section, we evaluate and compare the proposed localization algorithms. All localization algorithms are evaluated with and without body effect, as well as, for both static and mobile nodes. Table 2 details the acronyms used to identify the sample set acquired for each scenario. The sample set of atrium and lab scenarios is composed by 29 and 26 independent test runs, respectively. Each test run contains 200 position estimates. All the sample sets are created from real data.

The measurement noises for both the Taylor series and EKF methods are defined according to the statistics of noise with and without body influence.

### 3.1. Locating Static Devices without Body Influence

In the first experiment, the proposed positioning algorithms are evaluated for the atrium and lab scenarios, and without human body influence, respectively, the sample sets AWBS and LWBS. This experiment represents the best-case scenario since all the nodes are static and in LOS or under soft-NLOS. The soft-NLOS represents the propagation conditions when the direct path is blocked or partially blocked by machines, metallic cabinets or desk partitions.

**Figure 2:**
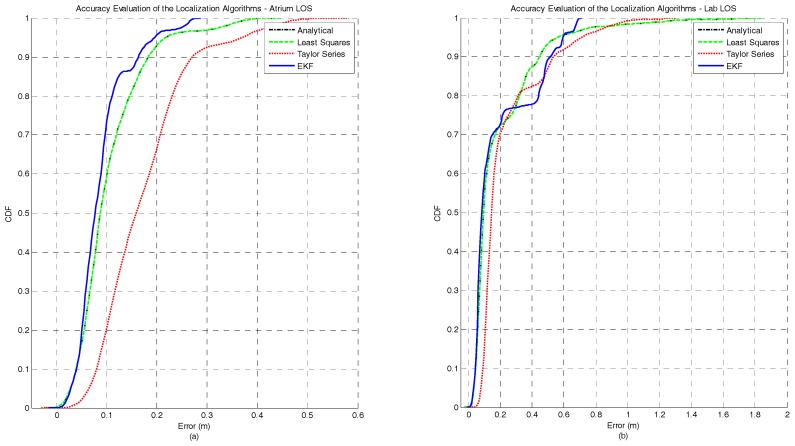
Comparison of the accuracy achieved by each algorithm for static nodes without human body interference. The localization algorithms were run in two different scenarios: (**a**) atrium and (**b**) lab.

Figure 2a,b represents the CDF of the accuracy error obtained for each positioning algorithm under the atrium and lab scenarios, respectively. It can be observed that for both the atrium and the lab scenarios—Figure 2—the EKF provides the lowest accuracy error. However, in the lab scenario a performance degradation of the EKF method can be observed between the percentiles 75 and 95. This performance degradation of the EKF method can be explained by the fact that the EKF is not a positioning algorithm but a tracking algorithm. So, the first position estimate for each independent test run is computed by the Analytical method. If the error in the first ranging measurements is high, coming position estimates, due to the EKF nature, will tend to be biased even if the ranging error is lower or inexistent. However, this strategy was adopted because in a real scenario the true position of the tag node is unknown, and the Analytical method is a good low-complexity candidate to provide the first position estimate. In Table 3, the complete figures of merit of the accuracy for all algorithms in both atrium and lab scenarios is presented.

**Figure 3:**
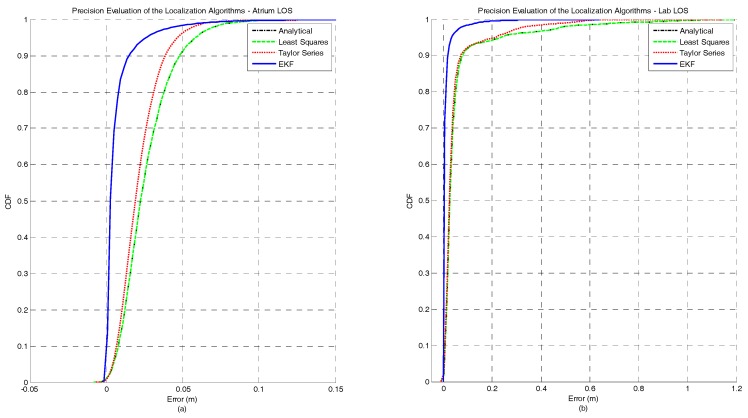
Comparison of the precision achieved by each algorithm for static nodes without human body interference. The localization algorithms were run in two different scenarios: (**a**) atrium and (**b**) lab.

Figure 3a,b represents the CDF of the precision error obtained for each positioning algorithm under atrium and lab scenarios, respectively. For both scenarios, the EKF method outperforms the other algorithms in terms of precision. As expected, the Taylor series method also shows a better performance when compared with the Least Squares and Analytical methods, +33% for the atrium and +46% for the lab. In Table 3, the complete figures of merit of the precision for all algorithms in both atrium and lab scenarios is presented.

Regarding the RMSE metric, the EKF method also performs better than the other algorithms for both scenarios. An interesting observation that can be made based on the RMSE metric is that the performance improvement of the EKF method is significantly higher for the atrium scenario (+20% and +90%) than in the lab scenario (+3% and +10%). This can be explained by the fact that the used noise statistics in the EKF were acquired in a corridor (free space area). Therefore, the atrium scenario best represents the used noise statistics of the signal than the lab scenario (where the signal propagation is under the influence of reflections, multipath and soft-NLOS phenomena). For the atrium scenario and for all algorithms, the X component of the RMSE is the predominant source of error in the net RMSE. On the other hand, for the lab scenario, the magnitude of both X and Y components are similar, but the Y component degrades up to 425% (for the EKF) whereas the X component degradation is 88% (also for the EKF). This increase in the RMSE of the Y component can be explained by the obstacles that create the soft-NLOS scenarios (machines, metallic cabinets or desk partitions), which are predominant in the Y axis. The Taylor Series is the method that experiences the lowest performance degradation when comparing both scenarios. However, it is also the method with the worst performance in both scenarios. The complete results for the RMSE metric can be consulted in Table 3.

**Figure 4:**
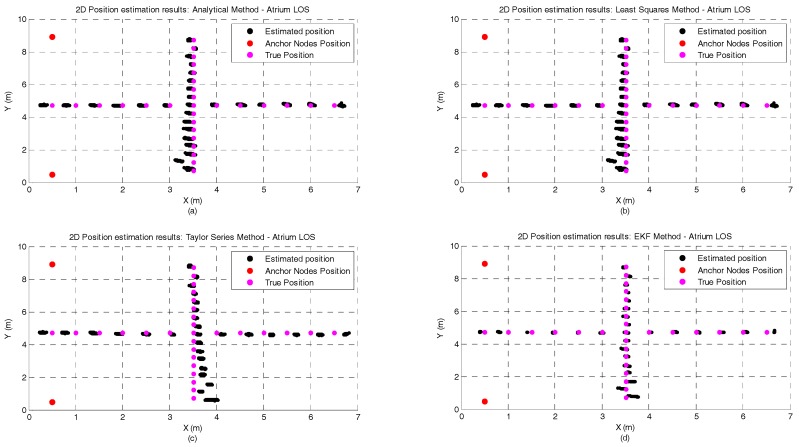
Graphical illustration of the 200 position estimates per test point in the atrium scenario and without human body interference. (**a**) Analytical method; (**b**) Least Squares method; (**c**) Taylor Series Method; and (**d**) EKF Method.

**Figure 5:**
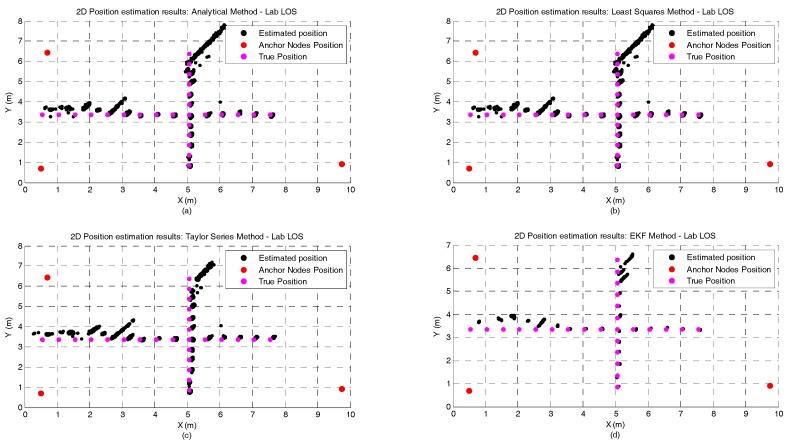
Graphical illustration of the 200 position estimates for each test point in the lab scenario and without human body interference. (**a**) Analytical method; (**b**) Least Squares method; (**c**) Taylor Series Method; and (**d**) EKF Method.

Figure 4 and Figure 5 illustrate the position estimates of each algorithm for atrium and lab scenarios, respectively. Through the analysis of both pictures, it is possible to confirm the gain in accuracy and more clearly in precision that the EKF has, in both scenario, when compared with the other positioning algorithms. The gain in precision is even more evident in the points where the ranging measurements are noisier (0 to 3 m in the X axis and 5 to 7 m in the Y axis, Figure 5). These points represent the soft-NLOS scenarios. From 0 to 3 m—in the X axis—the link anchor-tag is blocked by a metallic cabinet and from 5 to 7 m—in the Y axis—the link anchor-tag is blocked by a desk partition. In Figure 5 we can also see the positive bias created by the metallic cabinet.

### 3.2. Locating Static Devices with Body Influence

In the second experiment, the proposed positioning algorithms are evaluated for the same scenarios, but under the human body influence. In this evaluation, the positioning algorithms are run with the samples sets ABS and LBS for the Atrium and Lab scenarios, respectively. Additionally, the performance of the positioning algorithms is also evaluated when the NLOS identification and mitigation algorithm (proposed in Section 2.2) is applied for the ranging measurements, whose tag-anchor link is under the body influence.

**Figure 6:**
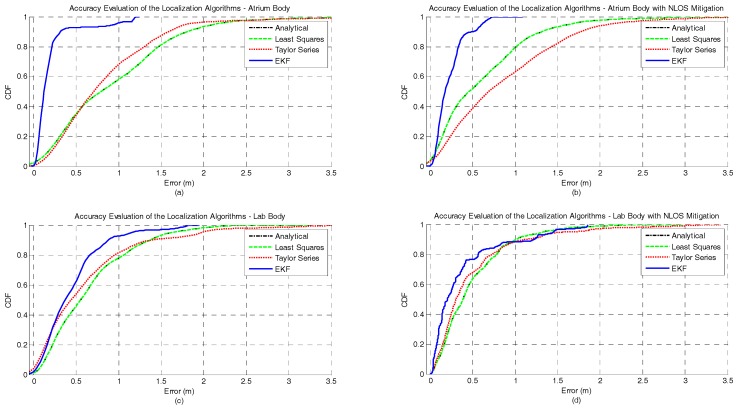
Comparison of the accuracy achieved by each algorithm for static nodes with human body interference and with or without NLOS mitigation. The first row represents the accuracy achieved by each algorithm in atrium with (**a**) and without (**b**) NLOS mitigation. Whereas, the second row represents the accuracy achieved by each algorithm in lab with (**c**) and without (**d**) NLOS mitigation.

The CDFs of the accuracy error under human body influence, in both atrium and lab scenarios, and with or without NLOS mitigation are illustrated in Figure 6. It can also be seen that the EKF method provides the lowest accuracy error for the majority of the scenarios studied. The only exception occurs when the EKF is combined with the NLOS mitigation algorithm in the lab scenario. For this scenario, the performance of the EKF method degrades after 85th percentile, making its performance worse than without NLOS mitigation algorithm and worse than the other three positioning algorithms. Another interesting observation is that unlike the other methods, the performance of the EKF in the 99th percentile is better in the atrium (0.7 m with NLOS mitigation and) than the lab (1.77 m without NLOS mitigation). The NLOS mitigation algorithm proposed in Section 2.2 proved to successfully improve the localization accuracy. This improvement in accuracy was more significant for the analytical and least squares methods. However, this error reduction is more evident for lower probabilities. The Taylor Series method showed the worst performance in both scenarios and with or without NLOS mitigation for the 99th percentile. In Table 4, the complete figures of merit of the accuracy for all algorithms in both atrium and lab scenarios and with or without NLOS mitigation is presented.

**Figure 7:**
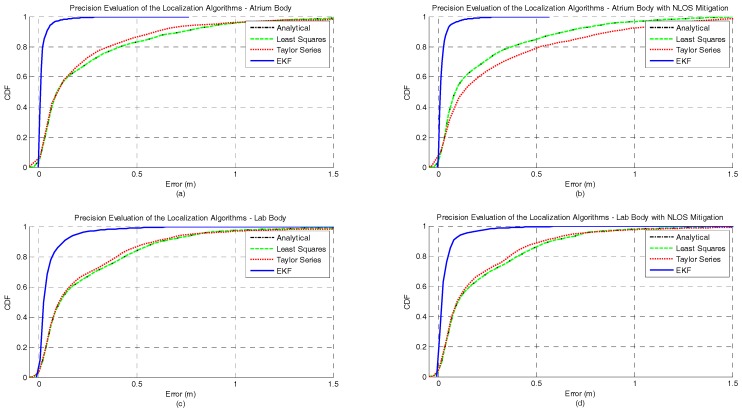
Comparison of the precision achieved by each algorithm for static nodes with human body interference and with or without NLOS mitigation. The first row represents the precision achieved by each algorithm in atrium with (**a**) and without (**b**) NLOS mitigation. Whereas, the second row represents the precision achieved by each algorithm in lab with (**c**) and without (**d**) NLOS mitigation.

The CDFs of the precision error under human body influence, in both atrium and lab scenarios, and with or without NLOS mitigation are illustrated in Figure 7. The best performance for both scenarios is achieved by the EKF method when the NLOS mitigation algorithm is run. For the atrium scenario no improvement is observed when the NLOS mitigation algorithm is applied whereas in the lab scenario an improvement of 29% is observed. In Table 4, the complete figures of merit of the precision for all algorithms in both atrium and lab scenarios, and with or without NLOS mitigation is presented.

Regarding the RMSE metric, the EKF method also outperforms the other methods in both scenarios. The net RMSE of all methods decreases when the NLOS mitigation is used. The only exception occurs with the Taylor Series method in the atrium, where the net RMSE slightly increased. Like in the previous experiment, the performance improvement of the EKF method is significantly higher for the atrium scenario. However, in this experiment is the Y component of the RMSE that is the predominant source of error in the net RMSE for the atrium. This happens because of the orientation of the human body during the measurement campaign, leading an error propagation in the Y axis. For the lab scenario, the magnitudes of X and Y components are similar. The complete results for the RMSE metric can be consulted in Table 4.

**Figure 8:**
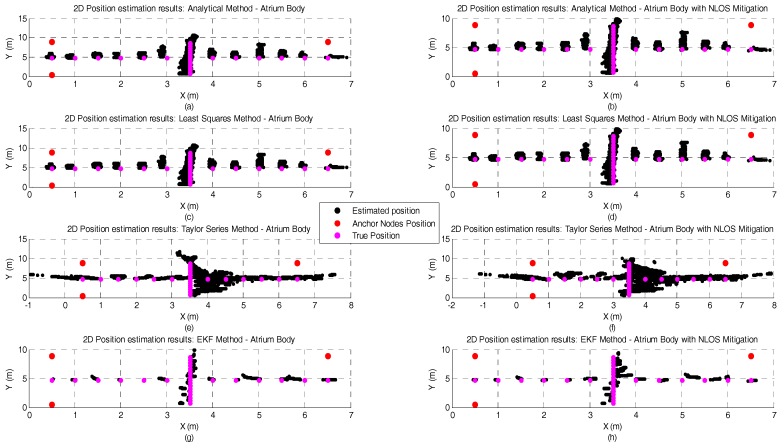
Graphical illustration of the 200 position estimates for each test point in the atrium scenario, with human body interference, and with or without NLOS mitigation. The plots on the left side of the figure represent the position estimates without NLOS mitigation and the plots on the right the position estimates with NLOS mitigation. Each row represents the results obtained by each positioning algorithm and their order from the top to the bottom of the figure is: Analytical method —(**a**,**b**); Least Squares method—(**c**,**d**); Taylor Series method—(**e**,**f**); and EKF Method—(**g**,**h**).

**Figure 9:**
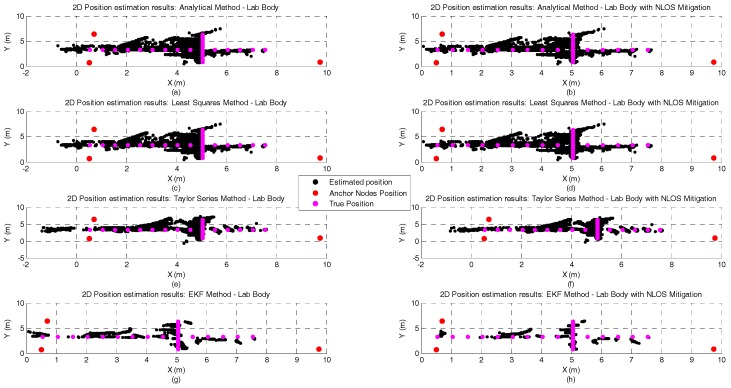
Graphical illustration of the 200 position estimates for each test point in the lab scenario, with human body interference, and with or without NLOS mitigation. The plots on the left side of the figure represent the position estimates without NLOS mitigation and the plots on the right the position estimates with NLOS mitigation. Each row represents the results obtained by each positioning algorithm and their order from the top to the bottom of the figure is: Analytical method —(**a**,**b**); Least Squares method—(**c**,**d**); Taylor Series method—(**e**,**f**); and EKF Method—(**g**,**h**).

Figure 8 and Figure 9 illustrate the position estimates of each algorithm with and without NLOS mitigation for atrium and lab scenarios, respectively. Through the analysis of both pictures, it is possible to confirm the gain in accuracy and more clearly in precision that EKF has, in both scenarios, when compared with the other positioning algorithms. Another interesting observation is the propagation of the ranging error due to the human body influence. From the analysis of both figures, especially for Analytical and Least Squares methods, it is possible to observe that the orientation of the human body has a clear effect on the error propagation. For the atrium scenario, the human body is perpendicular to the Y axis and the position error propagates in that direction. On the other hand, for the lab scenario, the human body is perpendicular to the X axis and the position error also propagates in that direction.

### 3.3. Locating Mobile Devices with Body Influence

In the third experiment, the proposed positioning algorithms are evaluated for mobile devices and under human body influence. In this evaluation, the positioning algorithms are run with the samples sets ABD and LBD for the Atrium and Lab scenarios, respectively. As in the previous test, the performance of the positioning algorithms is also compared when the NLOS identification and mitigation algorithm (proposed in Section 2.2) is applied to improve the ranging measurements.

**Figure 10:**
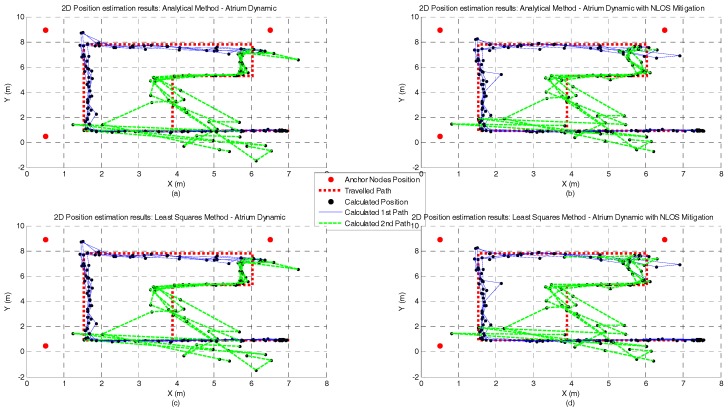
Atrium Dynamic test with Analytical (**a**,**b**) and Least Squares (**c**,**d**) methods and with and without NLOS identification and mitigation.

**Figure 11:**
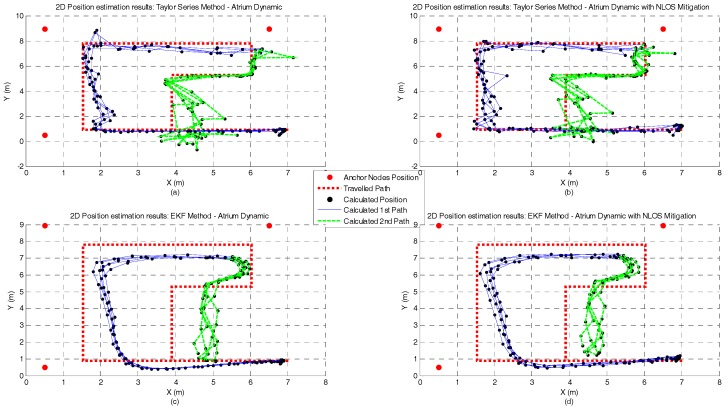
Atrium Dynamic test with Taylor Series (**a**,**b**) and EKF (**c**,**d**) methods and with and without NLOS identification and mitigation.

**Figure 12:**
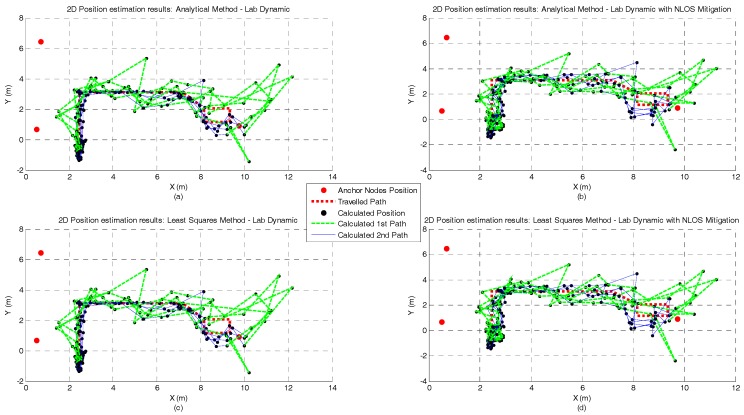
Lab Dynamic test with Analytical (**a**,**b**) and Least Squares (**c**,**d**) methods and with and without NLOS identification and mitigation.

**Figure 13:**
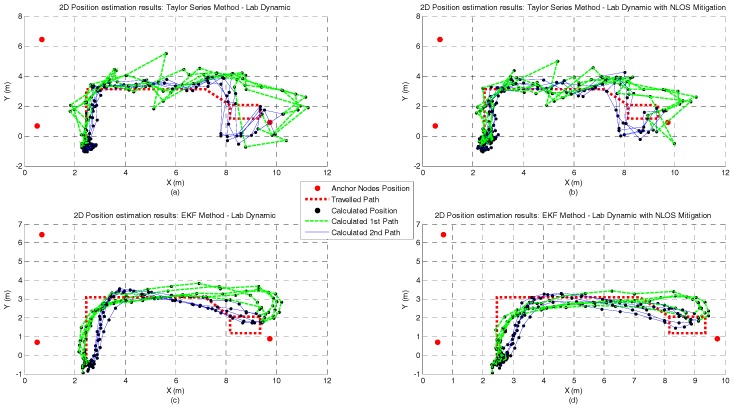
Lab Dynamic test with Taylor Series (**a**,**b**) and EKF (**c**,**d**) methods and with and without NLOS identification and mitigation.

Figure 10, Figure 11, Figure 12 and Figure 13 represent the position estimates and the user’s estimated path during the dynamic tests in atrium (Figure 10 and Figure 11) and lab (Figure 12 and Figure 13). On the left side of the figures (subplots a and c) are illustrated the position estimates without running the NLOS identification and error mitigation algorithm and, on the right side, the position estimates after running the NLOS identification and error mitigation algorithm (subplots b and d). For both scenarios, the walked path was designed so that the number of anchor nodes under NLOS condition varies between one and two. As can be seen from the figures, when two anchor nodes are in NLOS condition (green lines) the performance of all positioning algorithms severely deteriorates. When only one anchor node is under NLOS condition, the Analytical and the Least Squares methods perform better than the Taylor Series and the EKF methods. However, when the number of anchor nodes in NLOS condition increase, the performance of the Taylor Series and the EKF methods is higher. This is especially visible in the lab path where only the EKF method has a computed path close to the performed path. Another observation that can be drawn from the images is that the performance gain by running the NLOS identification and error mitigation algorithm is higher for the Taylor Series and EKF methods.

## 4. Discussion

UWB is an attractive way to perform localization, especially for IPSs that require an accurate position information and high measurement rate. The DW1000 UWB transceiver combines the UWB technology with ToA measurements, resulting in high accurate ranging estimates even in strong multipath environments. Additionally, it provides long range communication (300 m in LOS and 40 m in NLOS) and at an affordable price.

The EKF method persistently showed the best performance for all the evaluated metrics, for both atrium and lab scenarios and with or without human body influence. Although the gain in accuracy is evident, is the gain in precision that is really remarkable. For a 99th percentile, the precision reported by the EKF method in the worst scenario (lab under the body influence) can be three times lower than the second best performing algorithm. Whereas, for a 95th percentile, the precision reported is five times lower. Unlike the other methods, the EKF takes the previous estimates into account. This has a smoothing effect on the position estimation, making them more stable over the time, which can explain the higher performance of the EKF method.

By comparing the results of the different metrics for all algorithms under the two scenarios evaluated, the negative effect that the furniture and machines have on the performance of the positioning algorithms is clear. For the Analytical and Least Squares methods the accuracy worsens more than three times (from 0.36 to 1.22 m), the precision worsens more than nine times (from 0.08 to 0.72 m) and the RMSE more than two times (from 0.12 to 0.29 m). On the other hand, the Taylor Series and the EKF methods are more resilient to noisy measurements. Nevertheless, the accuracy degrades more than two times, the precision more than two times for the EKF and eight times for the Taylor Series method and the RMSE more than two times for the EKF and more than 50% for the Taylor Series. It is clear that the methods that account for the noise statistics achieve better results however at the cost of an increased complexity and computational requirements.

When body influence is considered, the performance of all algorithms worsens drastically. For the best performing algorithm (EKF) and for the best scenario (atrium), the accuracy error grows from 0.26 to 1.18 m when no mitigation algorithm is applied and to 0.7 m when the mitigation algorithm proposed is used. Another interesting observation is that the error reported in the lab is generally lower than the atrium. This can be explained by the positive impact of the multipath components created by objects and furniture in the lab scenario. When a human body blocks the direct path, it can completely block the RF propagation and the signal reaches the receiver through the phenomena of creeping waves [37]. However, these creeping waves have a lower propagation velocity, inducing an additional delay in the ToA estimation and therefore a higher error in the distance estimate. In such scenario, a multipath component due to a near object can be received first, resulting in a lower error.

Regarding the NLOS mitigation algorithm proposed, we can see that the overall performance of all algorithms was improved. The only exception occurred for the EKF method in the lab scenario, which can be explained by the used noise statistics. The noise statistics were obtained from the tests carried out in an open free space, which do not corresponds with the propagation conditions of the lab scenario. If the noise statistics of the lab scenario were used, a performance improvement is expected for the EKF method. However, this will make the performance of the IPS highly dependent on the lab scenario and this scenario does not represent all the propagation conditions for indoor environments, making the system scenario-dependent and, therefore, it does not comply with the emergency responders’ requirements. Although the accuracy of all methods is improved by running the NLOs mitigation algorithm—especially for the methods that do not use noise statistics—is the precision that is significantly improved (up to 50%).

Regarding the dynamic tests, the performance of all algorithms degrades as the number of anchor nodes under NLOS condition increases. As expected, the best trajectory is provided by the EKF method, which is especially visible in the lab scenario. However, when there is only one anchor node in NLOS condition, the performance of the Analytical and the Least Squares methods outperforms the EKF method. The lower performance of the EKF when compared with the other positioning algorithms can be explained by the low position update rate of the UWB systems (between 1 and 2 Hz).

Contrary to what was initially expected, the performance of the Taylor Series method was very unsatisfying. Especially if we account the increased complexity of this method when compared with the Analytical and the Least Squares methods. Its poor performance was even more evident when the body influence is accounted, in these scenarios the performance of the Taylor Series method was the worst. This poor performance can be explained by the initial estimate guess, obtained from the Analytical method. In the presence of noisy measurements, this first estimation will be too far from the true point, therefore, the algorithm will not reach the convergence, resulting in a poorer position estimate. A performance gain in the accuracy of the Taylor Series method can be obtained if the covariance matrix is tuned for each point. However, this will make the proposed IPS highly dependent on the evaluated scenario, which is against the requirements defined for emergency responders.

The performance of both Analytical and the Least Squares methods is the same in all considered scenarios. This was already expected as the only difference between methods is the mathematical formulation of the localization problem. The Analytical method is designed to locate tags with only three ranging measurements whereas the Least Squares method allows using more than three ranging measurements. When more than three ranging measurements are available, the performance of Least Squares method is expected to overcome the performance of the Analytical method.

Other important issues when selecting the localization algorithm of an IPS are its complexity and computational requirements. These parameters are important due to the necessary tradeoff between accuracy and energy consumption. However, since the application is for supporting emergency responders, it can be assumed that the IPS only needs to be operational for a few hours, so the complexity and computational requirements of the localization algorithm are not necessarily problems in our research as long as the gateway-PROTACTICAL has enough computational capabilities to run the positioning algorithm. Among all the considered algorithms, the EKF method is the one that requires more computational capabilities, followed by the Taylor Series, Least Squares and Analytical methods.

To keep the complexity of the localization algorithms a low as possible, in this study only the 2D position is computed. Since the main goal of knowing the 3D position is to identify which floor the emergency responder is, this information can be obtained from sensors like barometers and altimeters. Although the accuracy achieved by these sensors is lower than the UWB ranging measurements, it is enough to comply with the emergency responders’ requirements. Additionally, this design choice alleviates the deployment woes as the emergency responders have not to concern with the non-coplanarity of the anchor nodes—requirement for the 3D computation of the positioning algorithms.

Anchor placement is a very important issue in the algorithms’ performance that has not been addressed here. Although the performance of all positioning algorithms has been assessed using a minimum number of anchor nodes (three), the anchor nodes have been placed ensuring that there are always three non-collinear points. However, in a real deployment by emergency responders, the anchor nodes are more likely to be deployed in a trail topology. This different topology may affect the performance and, more important, the reliability of the positioning algorithms evaluated. Further tests are required to evaluate the impact that this topology may have on algorithms’ performance. A commonly used strategy to increase the accuracy and precision of the IPS is to add more anchor nodes. However, if the number of anchor nodes is increased, the complexity of the positioning algorithms will also increase, as well as, the energy consumption and the network latency and overhead.

## 5. Conclusions

In this paper, four ToA-based positioning algorithms were evaluated under different conditions (e.g., environments with different propagation conditions, static and dynamic target, and with or without NLOS influence due to the human body). Unlike the performance comparisons performed by other works, where tens of ranging measurements are used for the positioning process [38,39,40,41], in this work only three ranging measurements were used to assess the performance of the algorithms. This is especially important during the emergency responders’ missions, since the availability of radio signals is very low and there is a high demand for a high localization accuracy [2,4,6,8,24,55].

Among all the evaluated algorithms, the Extended Kalman Filter has shown the best results for all performance metrics in the static scenarios and when two anchor nodes are in NLOS condition for the dynamic tests. However, when only one anchor node is in NLOS condition, the Analytical and the Least Squares methods have a better performance in the dynamic scenarios. The weak performance of the Extended Kalman Filter for dynamic tests when only one node is in NLOS condition can be explained by the low position update rate of the UWB system (between 1 and 2 Hz), which affects the tracking capability. The NLOS identification and error mitigation algorithm also proved to be an effective tool to minimize the ranging error due to the human body. This was especially visible during the static tests.

## Figures and Tables

**Table 1:** Parameters used in the proposed NLOS Identification and Error Mitigation algorithm.

Parameter	Value
${\mathrm{th}}_{P_{D}}$	11.04 (dB)
$p_{1}$	0.007433
$p_{2}$	−0.06216
$p_{3}$	0.4843

**Table 2:** Acronyms used to refer the sample sets used for localization algorithms evaluation and comparison.

Test ID	Description of the Test
AWBS	Static localization without body influence (Case 1) in atrium scenario
ABS	Static localization with body influence (Case 2) in atrium scenario
ABD	Dynamic localization with body influence (Case 3) in atrium scenario
LWBS	Static localization without body influence (Case 1) in lab scenario
LBS	Static localization with body influence (Case 2) in lab scenario
LBD	Dynamic localization with body influence (Case 3) in lab scenario

**Table 3:** Performance comparison of the localization algorithms for the metrics accuracy, precision, and RMSE. All localization algorithms are compared for both atrium and lab scenarios and without human body interference.

Scenario	Accuracy (m)						Precision (m)						RMSE (m)		
	0	50	90	95	99	100	0	50	90	95	99	100	RMSEx	RMSEy	Net RMSE
**Analytic Method**															
AWBS	0	0.09	0.18	0.22	0.36	0.42	0	0.02	0.05	0.06	0.08	0.15	0.11	0.05	0.12
LWBS	0	0.09	0.45	0.56	1.22	1.81	0	0.03	0.08	0.23	0.72	1.18	0.19	0.22	0.29
**Least Squares Method**															
AWBS	0	0.09	0.18	0.22	0.36	0.42	0	0.02	0.05	0.06	0.08	0.15	0.11	0.05	0.12
LWBS	0	0.09	0.45	0.56	1.22	1.81	0	0.03	0.08	0.23	0.72	1.18	0.19	0.22	0.29
**Taylor Series Method**															
AWBS	0.02	0.16	0.27	0.37	0.46	0.54	0	0.02	0.04	0.05	0.06	0.18	0.17	0.09	0.19
LWBS	0.02	0.14	0.53	0.7	0.96	1.26	0	0.02	0.07	0.21	0.49	1.14	0.23	0.21	0.31
**EKF Method**															
AWBS	0	0.08	0.16	0.2	0.26	0.27	0	0	0.01	0.03	0.06	0.21	0.09	0.04	0.1
LWBS	0.02	0.08	0.52	0.59	0.68	0.68	0	0	0.02	0.03	0.14	0.64	0.17	0.21	0.28

**Table 4:** Performance comparison of the localization algorithms for the metrics accuracy, precision, and RMSE. All localization algorithms are compared for both atrium and lab scenarios, with human body interference, and with or without NLOS mitigation algorithm.

Scenario	Accuracy (m)						Precision (m)						RMSE (m)		
	0	50	90	95	99	100	0	50	90	95	99	100	RMSEx	RMSEy	Net RMSE
**Analytic Method**															
ABS	0.01	0.79	1.78	2.12	3.13	4.7	0	0.09	0.73	0.94	1.5	2.82	0.08	1.14	1.14
ABS_M	0	0.46	1.3	1.63	2.61	3.9	0	0.08	0.65	0.85	1.31	2.45	0.07	0.81	0.81
LBS	0.01	0.56	1.37	1.62	2.13	2.95	0	0.1	0.62	0.81	1.46	2.02	0.7	0.42	0.82
LBS_M	0	0.39	1.01	1.33	2.02	2.98	0	0.1	0.58	0.74	1.28	1.79	0.49	0.41	0.64
**Least Squares Method**															
ABS	0.01	0.79	1.78	2.12	3.13	4.7	0	0.09	0.73	0.94	1.5	2.82	0.08	1.14	1.14
ABS_M	0	0.46	1.3	1.63	2.61	3.9	0	0.08	0.65	0.85	1.31	2.45	0.07	0.81	0.81
LBS	0.01	0.56	1.37	1.62	2.13	2.95	0	0.1	0.62	0.81	1.46	2.02	0.7	0.42	0.82
LBS_M	0	0.39	1.01	1.33	2.02	2.98	0	0.1	0.58	0.74	1.28	1.79	0.49	0.41	0.64
**Taylor Series Method**															
ABS	0.04	0.69	1.61	1.74	3.38	6.56	0	0.08	0.6	0.91	2.4	5.83	0.61	0.88	1.07
ABS_M	0	0.67	1.72	2.07	3.17	4.83	0	0.12	0.88	1.18	1.6	4.43	0.71	0.84	1.1
LBS	0	0.43	1.36	1.99	3.14	3.67	0	0.09	0.59	0.78	1.82	2.9	0.6	0.65	0.88
LBS_M	0	0.3	1.13	1.59	2.92	3.42	0	0.09	0.53	0.72	1.49	2.97	0.49	0.55	0.73
**EKF Method**															
ABS	0.02	0.12	0.31	0.96	1.18	1.19	0	0.01	0.04	0.06	0.19	0.76	0.08	0.31	0.32
ABS_M	0.01	0.16	0.52	0.61	0.7	1.01	0	0.01	0.04	0.07	0.19	0.56	0.15	0.24	0.28
LBS	0.01	0.36	0.87	1.16	1.77	1.78	0	0.02	0.13	0.19	0.49	1.79	0.34	0.47	0.58
LBS_M	0	0.2	1.22	1.56	1.85	1.86	0	0.02	0.07	0.13	0.38	1.71	0.42	0.4	0.58
